# The Predictive Effects of Early Pregnancy Lipid Profiles and Fasting Glucose on the Risk of Gestational Diabetes Mellitus Stratified by Body Mass Index

**DOI:** 10.1155/2016/3013567

**Published:** 2016-02-15

**Authors:** Chen Wang, Weiwei Zhu, Yumei Wei, Rina Su, Hui Feng, Li Lin, Huixia Yang

**Affiliations:** ^1^Department of Obstetrics and Gynecology, Peking University First Hospital, Beijing 100034, China; ^2^National Institute of Hospital Administration, Beijing 100091, China

## Abstract

This study aimed at evaluating the predictive effects of early pregnancy lipid profiles and fasting glucose on the risk of gestational diabetes mellitus (GDM) in patients stratified by prepregnancy body mass index (p-BMI) and to determine the optimal cut-off values of each indicator for different p-BMI ranges. A retrospective system cluster sampling survey was conducted in Beijing during 2013 and a total of 5,265 singleton pregnancies without prepregnancy diabetes were included. The information for each participant was collected individually using questionnaires and medical records. Logistic regression analysis and receiver operator characteristics analysis were used in the analysis. Outcomes showed that potential markers for the prediction of GDM include early pregnancy lipid profiles (cholesterol, triacylglycerols, low-density lipoprotein cholesterol/high-density lipoprotein cholesterol ratios [LDL-C/HDL-C], and triglyceride to high-density lipoprotein cholesterol ratios [TG/HDL-C]) and fasting glucose, of which fasting glucose level was the most accurate indicator. Furthermore, the predictive effects and cut-off values for these factors varied according to p-BMI. Thus, p-BMI should be a consideration for the risk assessment of pregnant patients for GDM development.

## 1. Introduction

Gestational diabetes mellitus (GDM) is a common complication during pregnancy that occurs in 17.5% of Chinese pregnant women [1]. GDM leads to various adverse pregnancy outcomes not only during the perinatal phase but also in the long term [2, 3]. For example, women with GDM display an elevated risk for shoulder dystocia and the need for a caesarean section at birth, whereas the foetuses of women with GDM are prone to excessive intrauterine growth and neonatal hypoglycaemia. Furthermore, both women with GDM and their offspring experience a greater risk of obesity, type 2 diabetes, and cardiovascular disorders in the future.

Like type 2 diabetes, peripheral insulin resistance, combined with inadequate insulin secretion, plays the primary role in the pathophysiology of GDM. However, the exact aetiology of such insulin dysfunction is still not clearly understood. Investigators have indicated that lipid abnormalities serve as an inducing factor for insulin resistance [4]. Furthermore, the triglyceride to high-density lipoprotein cholesterol ratio (TG/HDL-C) has been confirmed as a clinical indicator of insulin resistance [5]. In addition, obesity is an independent risk factor for GDM, and the risk of GDM rises with an increase in the prepregnancy body mass index (p-BMI) [6]. All of these findings suggest that lipid profiles during early pregnancy may have an effect on the development of GDM, and this effect may vary due to p-BMI.

However, studies focused on the role of early pregnancy lipid profiles for the prediction of GDM are very limited and inconsistent [7, 8]. In China, in particular, lipid profile tests are not routinely requested at the first prenatal visit, and nearly half of all pregnant Chinese women do not undergo lipid profile testing during the first trimester.

Additionally, the fasting glucose level at the first prenatal visit has a strong ability to predict the incidence of GDM later in pregnancy [1]. Therefore, to clarify the predictive role of lipid profiles during early pregnancy and to compare the lipid prediction value with the fasting glucose prediction value, this study aimed to evaluate the predictive effect of lipid profiles [cholesterol, triacylglycerols, low-density lipoprotein cholesterol/high-density lipoprotein cholesterol ratio (LDL-C/HDL-C), and TG/HDL-C ratio] and fasting glucose levels during early pregnancy on the risk of GDM in groups stratified by p-BMI.

## 2. Materials and Methods

### 2.1. Data Source

The present analysis was part of a large retrospective study entitled “Systemic Random Sampling Survey On The Prevalence Of Gestational Diabetes Mellitus In Beijing (GDM prevalence survey, GPS).” In this study, a systemic cluster sampling method was used to identify 15 hospitals in Beijing, in which 15,194 pregnant women who delivered between June 20, 2013, and November 30, 2013, were included in the analysis. This study was reviewed and approved by the Institutional Review Boards of the Peking University First Hospital (reference number: 2013[578]). All participants provided written informed consent, and the ethics committee approved this consent procedure.

All participants in the retrospective study were eligible for the present analysis unless they met one or more of the following exclusion criteria: preexisting diabetes mellitus (DM) (209 patients), multiple births (253 patients), or missing data on early pregnancy lipid and fasting glucose concentrations (9,467 patients). Ultimately, a total of 5,265 participants were included.

### 2.2. Data Collection

A questionnaire was designed to collect information by interviewing all of the pregnant women who delivered during the study period and by reviewing their medical records from the day after they gave birth. The questionnaire was composed of two main sections: a demographic information section and a case data section. The demographic information section was completed during a face-to-face interview in the hospital room of the patient, whereas the case data section included information such as the glucose and lipid concentrations during early pregnancy and the dates on which they were evaluated as well as 75 g oral glucose tolerance test (OGTT) results. The information included in the latter section was reviewed and extracted from the medical records of each patient by the investigators. Prepregnancy weight was self-reported by each patient and was individually collected by our investigators.

The investigators at each hospital were trained before administering the survey. Each completed questionnaire was verified by an inspector. Data were coded and entered into a specially designed data software program that automatically flagged out-of-range values and logical mistakes. All questionnaires were administered by two individuals independently and were reviewed by a third person.

### 2.3. Definitions

*(1) GDM.* The GDM diagnostic criteria in this study followed the new criteria amended in August 2014 in China, which recommended that the diagnosis of GDM should be made when any one value met or exceeded a 0 h glucose level of 5.1 mM, a 1 h glucose level of 10.0 mM, and a 2 h glucose level of 8.5 mM after a diagnostic 75-g OGTT between the 24th and 28th week of gestation. A 0-h glucose level of 7.0 mM or a 2-h glucose level of 11.1 mM was considered sufficient to diagnose DM at any time [9], regardless of pregnancy stage.

*(2) Definition of Early Pregnancy.* It was defined as gestational age of less than 14 weeks.

*(3) Prepregnancy BMI (p-BMI).* Prepregnancy BMI was calculated as the prepregnancy weight (within three months before pregnancy) in kilograms divided by height in metres squared (kg/m^2^).

*(4) BMI Categories.* Classification of the BMI categories was based on the recommendations of the China Obesity Task Force of the Chinese Ministry of Health as follows: Underweight: BMI < 18.5 kg/m^2^; normal weight: 18.5 ≤ BMI < 24 kg/m^2^; overweight: 24 ≤ BMI < 28 kg/m^2^; obese: BMI ≥ 28 kg/m^2^ [10].

### 2.4. Statistical Analysis

We first divided the participants into two groups according to their 75-g OGTT results between 24 and 28 weeks of gestation, namely, the healthy group and the GDM group. We then evaluated the differences in the early pregnancy lipid profiles and fasting glucose concentrations between the women in the two groups. Then, based on the p-BMI categories of the participants (underweight: p-BMI < 18.5 kg/m^2^; normal weight: 18.5 ≤ p-BMI < 24 kg/m^2^; overweight: 24 ≤ p-BMI < 28 kg/m^2^; obese: p-BMI ≥ 28 kg/m^2^), we further divided the participants into four groups to assess the individual and joint value of early pregnancy lipid and fasting glucose levels for the prediction of GDM in patients stratified by p-BMI.

Data analysis was performed using the SAS 9.2 statistical software package (Peking University Clinical Research Institute). Continuous variables were expressed as the means ± standard deviation, and categorical variables were expressed as numbers and percentages. Differences in means between groups were assessed using the independent samples *t*-test, whereas Pearson's chi-square test was used for categorical variables. The threshold for statistical significance was set at 0.05. A multivariate binary logistic regression analysis was conducted to evaluate potential markers that were significantly associated with GDM after adjusting the effects of age and family history of DM. Receiver operating characteristic (ROC) analysis was performed using MedCalc software, and area under the curve (AUC) statistics were subsequently derived to determine the value of the individual factors and their combined ability to accurately discriminate the subjects of the GDM and non-GDM groups. A 95% confidence interval (CI) for the ROC analysis was calculated using the bootstrap method. The ROC analysis was also used to determine an optimal cut-off point for risk assessment (i.e., a cut-off yielding the best trade-off between sensitivity and specificity). In our study, we selected the optimal cut-off point at point at which the sum of the sensitivity and specificity was highest.

## 3. Results

A total of 5,265 pregnant women were recruited for our analysis. Compared with the excluded mothers (*n* = 9929), the included participants were more likely to have family history of DM (*p* < 0.05), but their age, p-BMI, education levels, and GDM incidence rate had no significant difference. The baseline characteristics of the study population were summarized in Table 1. Of the study population, 1,062 women (20.2%) were diagnosed with GDM. Age, p-BMI, and family history of DM were all significantly higher in women with GDM. Furthermore, early pregnancy serum fasting glucose, cholesterol, and triacylglycerol levels as well as TG/HDL-C and LDL-C/HDL-C ratios were significantly elevated in women with GDM compared with healthy pregnant women (*p* < 0.001) (Table 2).

Multivariate binary logistic regression analysis showed that, among women with a p-BMI ≥ 18.5 kg/m^2^, the odds ratio (OR) values of each lipid marker (cholesterol, triacylglycerols, TG/HDL-C ratio, and LDL-C/HDL-C ratio) increased as the p-BMI increased. Additionally, after adjusting for age and family history of DM, early pregnancy cholesterol concentrations were significantly related to the incidence of GDM in normal-weight (OR = 1.13, *p* = 0.02) and obese (OR = 1.55, *p* = 0.02) women; serum triacylglycerol level during early pregnancy was significantly associated with the prevalence of GDM in normal-weight (OR = 1.08, *p* = 0.04) and overweight (OR = 1.33, *p* = 0.01) women; the LDL-C/HDL-C ratio was only significantly associated with the risk of GDM in overweight women (OR = 1.42; *p* = 0.01). However, the TG/HDL-C ratio was a better predictor for GDM, as the associated OR value for the risk of GDM reached statistical significance in normal-weight (OR = 1.43, *p* < 0.001), overweight (OR = 1.63, *p* < 0.001), and obese (OR = 1.89, *p* = 0.03) women. However, fasting glucose levels in the first trimester appeared to be the best predictor for GDM. In each p-BMI category, fasting glucose levels were independently and significantly associated with the development of GDM later during pregnancy, and the OR values increased as the p-BMI level increased (underweight: OR = 2.93, *p* < 0.001; normal weight: OR = 3.15, *p* < 0.001; overweight: OR = 3.48, *p* < 0.001; obese: OR = 3.68, *p* < 0.001) (Table 3).

**Figure 1:**
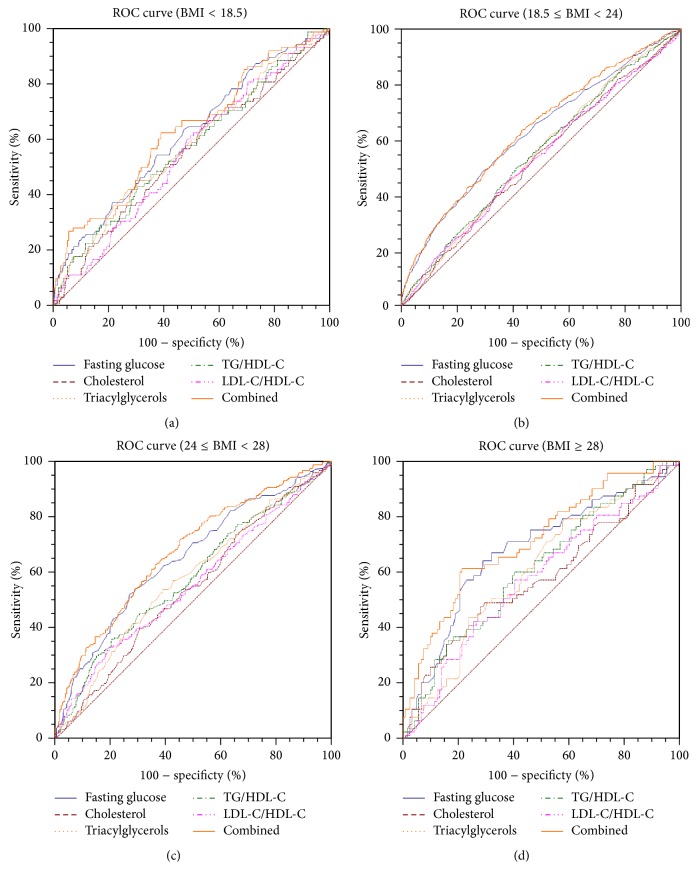
ROC curves of each factor and their combined ability to detect GDM, stratified by p-BMI. TG/HDL-C: triglyceride to high-density lipoprotein cholesterol ratio; LDL-C/HDL-C: low-density lipoprotein cholesterol/high-density lipoprotein cholesterol ratio.

Based on the ROC curves and AUC measurements, we determined the optimal cut-off values for early pregnancy fasting glucose, cholesterol, and triacylglycerol levels as well as for the TG/HDL-C and LDL-C/HDL-C ratios in each p-BMI stratification group, after which we analysed the value of combinations of these factors for the ability to predict GDM. All of these results are shown in Table 4 and Figure 1. After adjusting for age and family history of DM, we found that the optimal cut-off values for each indicator varied according to p-BMI level, and the cut-off values for cholesterol, TG/HDL-C ratio, and fasting glucose increased as the p-BMI increased. The cut-off value for early pregnancy fasting glucose was 4.77 mM in prepregnancy underweight women, 4.92 mM in prepregnancy normal-weight women, 5.00 mM in prepregnancy overweight women, and 5.05 mM in prepregnancy obese women. The respective cut-off values for underweight, normal-weight, overweight, and obese women were 4.18, 4.39, 4.84, and 4.90 mM for cholesterol, 1.16, 0.99, 1.40, and 1.09 mM for triacylglycerols, 0.63, 0.66, 1.07, and 1.20 for the TG/HDL-C ratio, and 0.89, 0.82, 0.52, and 0.57 for the LDL-C/HDL-C ratio. Most of the variables displayed a higher specificity than sensitivity. However, only the AUCs for the early pregnancy triacylglycerol level and TG/HDL-C ratio in the obese group surpassed 0.600, while the AUCs for early pregnancy fasting glucose level exceeded 0.600 in each p-BMI subgroup; that was 0.616 (95% confidence interval [CI], 0.578–0.652) for prepregnancy underweight women, 0.627 (95% CI: 0.611–0.644) for normal-weight women, 0.627 (95% CI: 0.611–0.644) for overweight women, and 0.682 (95% CI: 0.612–0.747) for obese women. We also generated ROC curves for all of the indicators together to observe their combined effects. The combined AUC was 0.630 (95% CI: 0.593–0.666) for prepregnancy underweight women, 0.638 (95% CI: 0.622–0.654) for normal-weight women, 0.676 (95% CI: 0.641–0.710) for overweight women, and 0.717 (95% CI: 0.648–0.770) for obese women.

## 4. Discussion

The results of this study suggest that, compared with normal pregnant women, women with GDM display significantly increased levels of cholesterol, triacylglycerols, and fasting glucose as well as elevated TG/HDL-C and LDL-C/HDL-C ratios during early pregnancy. Moreover, both of early pregnancy lipid profiles and fasting glucose can be potential markers for the prediction of GDM, and their predictive value improved as the p-BMI increased. However, fasting glucose values in early pregnancy appeared to be the best discriminator of GDM.

Changes in the early pregnancy lipid profiles in women with GDM were consistent with dyslipidaemia in obesity, which is characterized by elevated cholesterol, triacylglycerols, and LDL-C levels as well as decreased HDL-C levels [11]. These changes in lipid profiles are due to metabolic factors associated with insulin resistance [12], but the precise mechanisms for the associations between early pregnancy dyslipidaemia and the risk of GDM remain unclear. However, some aetiologies have been hypothesized. Some studies showed that excessive lipid accumulation may lead to an elevated oxidative stress, which correlates with insulin resistance [13], whereas other studies have shown that abnormal lipid metabolism can lead to the direct destruction of the function of *β* cells of the pancreas [14]. In addition, obesity not only is an important risk factor for GDM but also exerts a critical influence on lipid profiles, which supports our hypothesis that lipid profiles during early pregnancy can predict the development of GDM, though their predictive effects are not very strong in our study.

Numerous studies have demonstrated a relationship between dyslipidaemia and glucose intolerance as well as type 2 diabetes [15, 16]; however, studies focused on the assessment of the association between early pregnancy lipid profiles and GDM risk are limited and inconsistent. Enquobahrie et al. found that women with plasma triacylglycerol levels ≥ 137 mg/dL displayed a 3.5-fold increased risk of GDM, and each 20 mg/dL increase in triacylglycerol levels led to a 10% increase in GDM risk; however, no other associations could be observed between lipid changes and GDM risk [7]. dos Santos-Weiss and colleagues obtained similar results after conducting a matched case-control study. They noted that the TG/HDL-C ratio and triacylglycerol levels between 12 and 13 weeks of gestation were good indicators for the prediction of GDM; however, the TG/HDL-C ratio was a much better predictor [17]. In contrast, another study indicated that only low HDL-C concentrations had a significant and independent role for the prediction of GDM [18].

In our study, the results suggest that the potential for each lipid index to predict GDM varied according to p-BMI. However, the inconsistency may also be due to confounding factors or insufficient sample size. Moreover, we observed that the prevalence odds for each lipid index for the prediction of GDM presented an approximate linear trend between groups. Although some of the OR values were not significant, our data show that patients with higher p-BMI values show a more significant association between early pregnancy lipid profiles and GDM incidence. This point was also demonstrated based on our ROC curve analysis. The AUC values for each lipid marker increased in conjunction with p-BMI. Moreover, similar results were observed for early pregnancy fasting glucose levels and the combined value of fasting glucose levels and lipid markers for the prediction of GDM.

Early pregnancy fasting glucose was the most accurate predictor for the development of GDM according to our analysis, which is in accordance with previous studies. Harrison et al. indicated that, after adjusting for confounders, only elevated fasting glucose level but not elevated triacylglycerol level or decreased HDL-C level during early pregnancy was significantly associated with GDM development. The OR for fasting glucose and GDM risk was 10.03 according to the International Association for Diabetes in Pregnancy Study Group (IADPSG) GDM diagnostic criteria, and the AUC was 0.83 (95% CI: 0.77–0.90) [19].

In our present analysis, significant associations between increased early pregnancy fasting glucose level and the risk of developing GDM was present for each p-BMI subgroup. A previous study by our group evaluated the value of fasting glucose testing at the first prenatal visit for the diagnosis of GDM in China, and the results demonstrated that fasting glucose values at the first prenatal visit strongly correlated with the development of GDM (Pearson's *χ* ^2^ = 959.3, *p* < 0.001). Furthermore, Zhu et al. proposed that, for women with fasting glucose levels between 5.10 mM and 6.09 mM at the first prenatal visit, a healthy lifestyle intervention was needed for the prevention of GDM [1].

However, when we tried to improve the predictive ability of early pregnancy lipid profiles and fasting glucose by combining them as a joint risk assessment tool, we found that, in each p-BMI stratification group, using lipid profiles and fasting glucose together just gave a little higher AUC than using fasting glucose as the only predictor for GDM. That is to say, adding early pregnancy lipid profiles to fasting glucose could not dramatically improve its ability to detect GDM. Thus, early pregnancy fasting glucose should still be a significant risk factor that needs to be early screened for effective identification of GDM risk.

Another major contribution from our study was determining the optimal cut-off values for each lipid marker and fasting glucose for the prediction of GDM according to p-BMI. To the best of our knowledge, this is the first study to evaluate the optimal cut-off value for predicting GDM according to the four p-BMI categories. Of note, the cut-off value for each indicator analysed in this study varied according to the p-BMI category, and the cut-off values of the cholesterol level, TG/HDL-C ratio, and fasting glucose level increased as the p-BMI increased. For example, the cut-off values for the TG/HDL-C ratio were 0.63, 066, 1.07, and 1.20 for prepregnancy underweight, normal-weight, overweight, and obese women, respectively. Although the sensitivity and specificity for each lipid marker were not very high, our results indicate that p-BMI is an important factor which should be considered when using early markers to predict GDM. Thus, it is necessary to examine the laboratory results of pregnant women in the context of their p-BMI to determine GDM risk.

Few studies have evaluated the cut-off values of the lipid markers for the prediction of GDM. dos Santos-Weiss et al. noted that when defining the cut-off point of the TG/HDL-C ratio between 12 and 23 weeks of gestation as 0.099, the sensitivity and specificity reached 82.6% and 83.4%, respectively (AUC 0.886, *p* < 0.0001) [17]. Another study performed in Chinese patients established the cut-off value for the TG/HDL-C ratio between gestation weeks 24 and 28 with respect to the detection of GDM at 1.12 (AUC 0.617, 95% CI: 0.548–0.686) [20]. In our study, we restricted the definition of early pregnancy to before gestation week 14 and analysed the optimal lipid and fasting glucose cut-off values in patients divided into four p-BMI categories. Therefore, our study was more rigorously structured than previous studies.

This study was based on a systemic cluster sampling survey and was rationally designed. Additionally, our study was conducted by trained staff, and most of the items included in the questionnaire were based on medical records. This approach ensured the standardization of data collection. Furthermore, our analysis yielded details for patients in each of the four p-BMI categories. However, several limitations of this study should be noted. First, our study was retrospective in nature, and the p-BMI values were self-reported; therefore, group classification errors are possible due to recall bias. Second, lipid profiles and fasting glucose levels are closely related to lifestyle. However, because we did not consider lifestyle factors in our analysis, patient lifestyle could be a confounding factor for some of our analyses. Third, the sample size included in our study may still have been insufficient. Moreover, in this study, we focused on Chinese singleton, nondiabetic, pregnant women; therefore, our results may not be generalizable to the overall population.

In conclusion, our study showed that women with GDM had significantly increased cholesterol levels, triacylglycerol levels, TG/HDL-C ratios, LDL-C/HDL-C ratios, and fasting glucose levels during early pregnancy. Moreover, early pregnancy cholesterol levels, triacylglycerol levels, TG/HDL-C ratios, LDL-C/HDL-C ratios, and fasting glucose levels were associated with GDM risk, while fasting glucose appeared to be the most accurate predictor of GDM. Besides, as the optimal cut-off value for each indicator analysed in this study varied according to p-BMI, and the predictive value of these factors increased in significance along with p-BMI, thus, p-BMI should be considered when using early markers to predict GDM. However, several problems still need to be addressed, such as determining the optimal time during which lipid markers most accurately predict GDM development and determining the precise mechanism between dyslipidaemia and GDM. Thus, future clinical trials and basic research studies should be conducted to address these questions.

## Figures and Tables

**Table 1:** Maternal baseline characteristics.

	Healthy	GDM	*P*
	*N* = 4203	*N* = 1062	
Age (years)	28.29 ± 3.79	29.46 ± 3.96	<0.001
p-BMI (kg/m^2^)	21.33 ± 3.03	22.52 ± 3.36	<0.001
<18.5 (%)	621 (14.90)	89 (8.44)	<0.001
18.5–23.9 (%)	2896 (69.48)	645 (61.14)	<0.001
24–27.9 (%)	522 (12.52)	245 (23.22)	<0.001
≥28 (%)	129 (3.10)	76 (7.20)	<0.001
Family history of diabetes (%)	608 (14.47)	253 (23.82)	<0.001
Education level			
Graduate and above (%)	616 (14.64)	143 (13.47)	0.343
College/university (%)	2717 (64.64)	723 (68.08)	0.036
High school (%)	517 (12.30)	136 (12.81)	0.655
Junior high school and below (%)	308 (7.32)	54 (5.09)	0.003

GDM: gestational diabetes mellitus; p-BMI: prepregnancy body mass index.

**Table 2:** Maternal early pregnancy fasting glucose and lipid profiles.

	Healthy	GDM	*P*
	*N* = 4203	*N* = 1062	
Early pregnancy			
Cholesterol (mM)	4.44 ± 0.78	4.57 ± 0.83	<0.001
Triacylglycerols (mM)	1.17 ± 0.91	1.38 ± 2.16	<0.001
TG/HDL-C ratio	0.71 ± 0.46	0.92 ± 1.61	<0.001
LDL-C/HDL-C ratio	1.38 ± 0.52	1.51 ± 0.61	<0.001
Fasting glucose (mM)	4.73 ± 0.39	4.96 ± 0.45	<0.001
Lipid detection at weeks' gestation	10.76 ± 2.57	10.70 ± 2.59	0.635
Fasting glucose detection at weeks' gestation	10.83 ± 2.86	10.77 ± 2.94	0.499

GDM: gestational diabetes mellitus; TG/HDL-C: triglyceride to high-density lipoprotein cholesterol ratio; LDL-C/HDL-C: low-density lipoprotein cholesterol/high-density lipoprotein cholesterol ratio.

**Table 3:** Binary logistic regression analysis of the risk of GDM stratified by prepregnancy BMI.

p-BMI categories	Cholesterol			Triacylglycerols			TG/HDL-C ratio			LDL-C/HDL-C ratio			Fasting glucose		
	OR	95% CI	*P*	OR	95% CI	*P*	OR	95% CI	*P*	OR	95% CI	*P*	OR	95% CI	*P*
<18.5	1.22	(0.93–1.61)	0.15	1.28	(0.95–1.73)	0.10	1.49	(0.93–2.42)	0.10	1.38	(0.97–1.96)	0.07	2.93	(1.62–5.27)	<0.001
18.5–23.9	1.13	(1.02–1.26)	0.02	1.08	(1.01–1.16)	0.04	1.34	(1.15–1.58)	<0.001	1.17	(1.00–1.37)	0.05	3.15	(2.51–3.94)	<0.001
24–27.9	1.17	(0.96–1.42)	0.12	1.33	(1.09–1.67)	0.01	1.63	(1.25–2.14)	<0.001	1.42	(1.11–1.83)	0.01	3.48	(2.34–5.18)	<0.001
≥28	1.55	(1.08–2.22)	0.02	1.49	(0.93–2.39)	0.10	1.89	(1.08–3.29)	0.03	1.51	(0.93–2.43)	0.09	3.68	(1.83–7.33)	<0.001

p-BMI: prepregnancy body mass index; TG/HDL-C: triglyceride to high-density lipoprotein cholesterol ratio; LDL-C/HDL-C: low-density lipoprotein cholesterol/high-density lipoprotein cholesterol ratio.

**Table 4:** The optimal cut-off value for each factor and their combined value for the detection of GDM stratified by p-BMI.

	p-BMI categories kg/m^2^	Cut-off value mM	AUC	95% CI	Sensitivity	Specificity
Fasting glucose	<18.5	4.77	0.616	0.578, 0.652	54.5	62.7
	18.5–23.9	4.92	0.627	0.611, 0.644	49.5	70.3
	24–27.9	5.00	0.627	0.611, 0.644	52.3	72.8
	≥28	5.05	0.682	0.612, 0.747	64.0	71.3

Cholesterol	<18.5	4.18	0.554	0.517, 0.592	66.3	45.2
	18.5–23.9	4.39	0.533	0.517, 0.550	55.5	51.4
	24–27.9	4.84	0.533	0.517, 0.550	39.6	69.7
	≥28	4.90	0.584	0.511, 0.654	49.3	69.5

Triacylglycerols	<18.5	1.16	0.582	0.544, 0.619	41.6	73.6
	18.5–23.9	0.99	0.552	0.535, 0.568	61.9	46.4
	24–27.9	1.40	0.552	0.535, 0.568	49.4	65.5
	≥28	1.09	0.610	0.537, 0.679	80.3	38.8

TG/HDL-C ratio	<18.5	0.63	0.572	0.535, 0.609	43.8	67.9
	18.5–23.9	0.66	0.554	0.537, 0.571	50.2	58.9
	24–27.9	1.07	0.554	0.537, 0.571	36.3	79.8
	≥28	1.20	0.615	0.543, 0.684	36.0	84.5

LDL-C/HDL-C ratio	<18.5	0.89	0.555	0.517, 0.592	65.2	46.7
	18.5–23.9	0.82	0.536	0.519,0.553	65.2	50.4
	24–27.9	0.52	0.536	0.519, 0.553	33.1	80.4
	≥28	0.57	0.579	0.506, 0.649	57.3	61.2

Combined	<18.5		0.630	0.593, 0.666		
	18.5–23.9		0.638	0.622, 0.654		
	24–27.9		0.676	0.641, 0.710		
	≥28		0.717	0.648, 0.77		

p-BMI: prepregnancy body mass index; TG/HDL-C: triglyceride to high-density lipoprotein cholesterol ratio; LDL-C/HDL-C: low-density lipoprotein cholesterol/high-density lipoprotein cholesterol ratio.
